# Downy mildew disease promotes the colonization of romaine lettuce by *Escherichia coli* O157:H7 and *Salmonella enterica*

**DOI:** 10.1186/s12866-015-0360-5

**Published:** 2015-02-04

**Authors:** Ivan Simko, Yaguang Zhou, Maria T Brandl

**Affiliations:** Crop Improvement and Protection Research Unit, U.S. Department of Agriculture, Agricultural Research Service, Salinas, CA 93905 USA; Produce Safety and Microbiology Research Unit, U.S. Department of Agriculture, Agricultural Research Service, Albany, CA 94563 USA

**Keywords:** Produce contamination, *Bremia lactucae*, Plant pathogen, Oomycete, Human pathogen, Foodborne pathogen, Enteric pathogen, Basal plant immunity

## Abstract

**Background:**

Downy mildew, a plant disease caused by the oomycete *Bremia lactucae*, is endemic in many lettuce-growing regions of the world. Invasion by plant pathogens may create new portals and opportunities for microbial colonization of plants. The occurrence of outbreaks of *Escherichia coli* O157:H7 (EcO157) and *Salmonella enterica* Typhimurium (*S*. Typhimurium) infections linked to lettuce prompted us to investigate the role of downy mildew in the colonization of romaine lettuce by these human pathogens under controlled laboratory conditions.

**Results:**

Whereas both EcO157 and *S.* Typhimurium population sizes increased 10^2^-fold on healthy leaf tissue under conditions of warm temperature and free water on the leaves, they increased by 10^5^-fold in necrotic lesions caused by *B. lactucae*. Confocal microscopy of GFP-EcO157 in the necrotic tissue confirmed its massive population density and association with the oomycete hyphae. Multiplication of EcO157 in the diseased tissue was significantly lower in the RH08-0464 lettuce line, which has a high level of resistance to downy mildew than in the more susceptible cultivar Triple Threat. qRT-PCR quantification of expression of the plant basal immunity gene PR-1, revealed that this gene had greater transcriptional activity in line RH08-0464 than in cultivar Triple Threat, indicating that it may be one of the factors involved in the differential growth of the human pathogen in *B. lactucae* lesions between the two lettuce accessions. Additionally, downy mildew disease had a significant effect on the colonization of EcO157 at high relative humidity (RH 90-100%) and on its persistence at lower RH (65-75%). The latter conditions, which promoted overall dryness of the lettuce leaf surface, allowed for only 0.0011% and 0.0028% EcO157 cell survival in healthy and chlorotic tissue, respectively, whereas 1.58% of the cells survived in necrotic tissue.

**Conclusions:**

Our results indicate that downy mildew significantly alters the behavior of enteric pathogens in the lettuce phyllosphere and that breeding for resistance to *B. lactucae* may lower the increased risk of microbial contamination caused by this plant pathogen.

## Background

**Figure 1:**
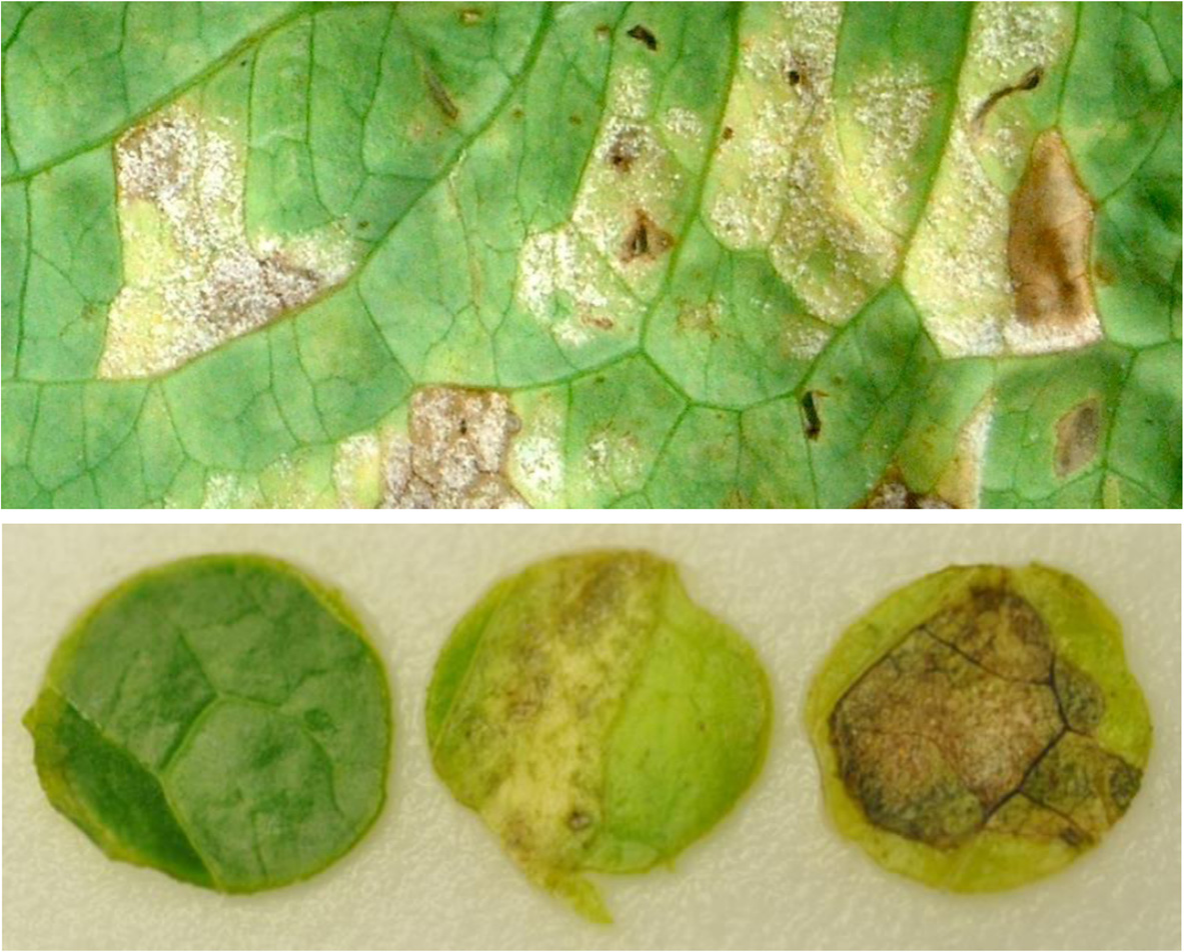
**Photographs illustrating in the upper panel, typical downy mildew disease symptoms on the abaxial lettuce leaf surface with chlorotic and necrotic tissue, and white/grey spores of** ***B. lactucae*** **hyphae emerging from the infected tissue.** In the lower panel, typical leaf discs of healthy, chlorotic, and necrotic (left to right) tissue of romaine lettuce tissue cultivar Green Towers used as samples for measurement of EcO157 population sizes in our study.

Contamination of lettuce with the human enteric pathogens, *Escherichia coli* O157:H7 and *Salmonella enterica* has caused several outbreaks of foodborne disease in the US and other parts of the world [1,2]. Although the persistence of these human pathogens on lettuce in the field has been documented [3-6], factors that contribute to their survival and potential multiplication on plants remain largely unknown. As for other bacteria that immigrate onto plant surfaces, it is likely that the survival of enteric pathogens is affected by the plant microbial community and various physicochemical stresses that prevail in the lettuce phyllosphere. In addition to moving through stomata to the mesophyll tissue of the leaf [7], where it may be shielded from such conditions as desiccation and UV irradiation, EcO157 may gain access to protective sites that result from the infection by plant pathogens. Lettuce can be infected by a broad range of bacterial, fungal, oomycete, and viral pathogens [8], the prevalence of which greatly depends on environmental conditions and the genotype of the plant itself. Downy mildew, a disease of lettuce caused by the obligate oomycete pathogen, *Bremia lactucae* Regel, is endemic to many important lettuce producing areas of California [9]. Several outbreaks of EcO157 infections have been traced back to a major lettuce-production region in California [2] in which this enteric pathogen is highly prevalent [10]. *B. lactucae* survives in crop debris from infected leaf tissue and on weed hosts. Representative symptoms of downy mildew disease on lettuce are shown in Figure 1. In the early stages of plant infection, the pathogen causes angular chlorotic lesions bordered by the leaf veins. Growth of mycelia and the presence of small dense masses of grayish spores are observed mostly on the abaxial surface of the leaves. The infected tissue eventually becomes necrotic and dies [8]. *B. lactucae* benefits from humidity and cool temperatures and therefore, infection rates of lettuce are high during conditions promoting long durations of morning leaf wetness [11]. The incidence of the disease in the Salinas growing region of Coastal California generally increases in the autumn lettuce crop [12].

Biotrophic and necrotrophic fungi that cause post-harvest decay have been shown in several instances to enhance the colonization of fruit and vegetables by enteric pathogens. *Glomerella cingulata* infection of apple fruit promoted proliferation of *Listeria monocytogenes* and EcO157, likely due to a change of pH from 4.0 to 7.0, whereas the reverse effect was observed with *Penicillium expansum* [13,14]. *Alternaria alternata* and *Cladosporium* spp. had a positive effect on *S. enterica* colonization of tomato fruit [15] and *Fusarium* spp. prolonged the survival of EcO157 on tomato under storage conditions but not that of *L. monocytogenes* [16]. These observations suggest that the effect of pathogenic fungi on enteric pathogens in plant tissue may vary depending on several factors in this tri-partite association.

Despite the endemic nature of downy mildew disease in lettuce fields in California and other regions in the United States, and the increased appearance of disease symptoms on plants near harvest maturity i.e. not long prior to the lettuce crop reaching the consumer, the role of *B. lactucae* infection in the behavior of EcO157 on lettuce has not been investigated. *B. lactucae* requires free water on the phylloplane for spore germination and invasion of plant cells, a condition that also promotes the survival and multiplication of EcO157 on lettuce [17,18]. Additionally, lesions caused by *B. lactucae* are known to act as portals to necrotrophic plant pathogens, such as *Botrytis cinerea,* which colonize the broken tissue as secondary invaders [19]. Our study investigates the potential of downy mildew lesions to serve as a portal for EcO157 and to create a habitat where the human pathogen may thrive opportunistically by gaining protection from environmental insults within the plant tissue.

## Results and discussion

### Effect of downy mildew disease on EcO157 and *S. enterica* under wet conditions

**Figure 2:**
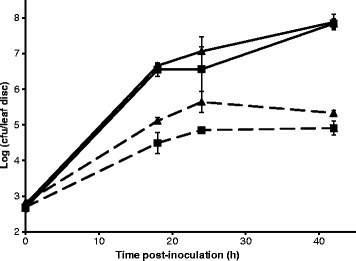
**Effect of downy mildew on EcO157 and** ***S.*** **Typhimurium population dynamics on romaine lettuce (cultivar Green Towers) leaves over time after inoculation and incubation of the leaves under conditions of warm temperature and presence of free water on the leaves.** Population sizes of EcO157 (▲) and *S.* Typhimurium (■) were assessed on leaf discs of healthy (dotted lines) and *B. lactucae*-infected necrotic (solid lines) lettuce tissue. Data points represent the mean of log-10 (cfu per disc) for three replicate discs sampled from different leaves. Error bars indicate standard error of the mean.

In order to investigate the growth potential of EcO157 and *S. enterica* in diseased tissue during presence of free water on lettuce plants, such as may occur during periods of rain, dew or overhead watering, the pathogens were inoculated onto leaves that were then incubated under conditions promoting wetness on their surface. As we observed previously on romaine lettuce [17], both enteric pathogens achieved population increases of approximately 10^2^-fold on the healthy leaf tissue (Figure 2). The presence of necrosis due to infection by *B. lactucae* (Figure 1) promoted enhanced colonization by both enteric bacteria since the population sizes on the healthy and infected tissue diverged greatly by 18 h post-inoculation. Although we did not test for the possible asymptomatic presence of the oomycete in some of the visually healthy tissue that was used in our study, the disease symptoms clearly resulted in greater population densities of the enteric pathogens than the seemingly healthy tissue. Overall, the population sizes of EcO157 and *S.* Typhimurium in the lesions increased at least 10^5^-fold over 42 h at warm temperature. This high carrying capacity of the diseased leaf tissue is likely due to the abundant nutrients made available by the oomycete degradation of plant cells and their contents.

Lettuce leaves are rich in a variety of carbohydrates, including sucrose, glucose, fructose, galactose and mannose [20,21]. EcO157 rapidly activates multiple carbohydrate transport systems and utilization pathways upon exposure to lysates of romaine lettuce leaves [22]. Although it is likely that the oomycete itself acquires a large share of the nutrients present in plant cells, the collapsed leaf tissue at advanced stages of the disease such as that inoculated in this study probably supports high rates of multiplication of the human pathogens without overall significant competition by the plant pathogen. Another possibility is that at advanced stages of the disease, the oomycete scavenges plant components, the degradation of which makes substrates available to EcO157. It is noteworthy that leaf damage caused by phytopathogens does not *de facto* promote colonization by enteric pathogens. For example, *S. enterica* levels in lettuce shoots also inoculated with lettuce mosaic virus (LMV) were not different than in non-LMV-infected plants and even decreased in infected plants grown under water stress [23]. Additionally, EcO157 cell density on spinach was unaffected by its co-inoculation with *Pseudomonas syringae* DC3000, a plant pathogen that caused necrotic lesions on the leaves [24]. Thus, factors additional to leakages of substrates from infected cells are at play in the interaction of enteric pathogens with plant pathogens.

### Localization of EcO157 by microscopy

**Figure 3:**
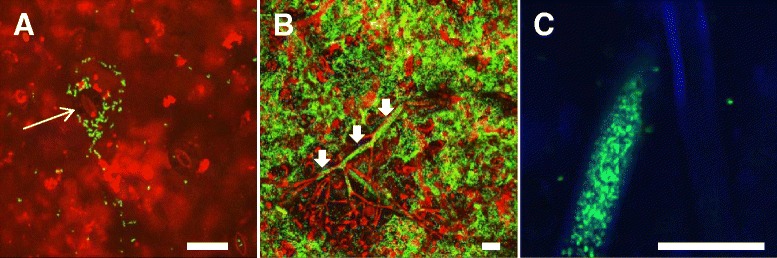
**Confocal micrographs of GFP-EcO157 cells colonizing romaine lettuce leaf tissue (cultivar Green Towers). (A)** Sparse colonization of healthy tissue by EcO157 with greater proliferation around a stomate (long arrow). **(B)** High density of EcO157 cells in the necrotized tissue due to infection with the plant pathogen *B. lactucae*, and association of the human pathogen with the oomycete hyphae (short arrows). The large masses of GFP-cells are evidenced by the green fluorescent signal. The yellow signal results from the GFP fluorescence of the bacterial cells co-localizing with the red autofluorescence of the leaf or of the oomycete. **(C)** Single optical scan longitudinally across a hypha revealing that the GFP-EcO157 cells invaded the oomycete tissue either actively, or passively via damages to the hyphal wall. In contrast, the hypha to the right remained uncolonized. In this image, the signal acquired in the red and far red was pseudo-colored blue. The left and middle images are pseudo-3D projections of multiple optical slices in the *z* range. Bars, 20 μm.

Confocal microscopy of downy mildew necrotic lesions clearly illustrates the large masses of GFP-EcO157 cells that result from colonization of these sites compared with the few microcolonies that formed at discrete locations on the epidermis of healthy leaf tissue (Figure 3A). Of interest also, is the external and internal colonization of the oomycete hyphae that we observed 40 h after EcO157 came in contact with the plant pathogen through inoculation of the necrotic tissue. This is apparent in Figure 3B but more specifically in the single confocal optical scan through the hypha shown in Figure 3C, which reveals that the mass of EcO157 cells is bound by the hyphal cell wall and located internally. It remains unclear however, if the human pathogen invaded the oomycete actively, or if it gained access due to damage and thus, ports of entry, on the hyphae. This important aspect of EcO157-*B. lactucae* interactions deserves further investigation, particularly in the light of previous studies demonstrating the inhibition of fungi by various bacterial species [25]. If invasion of hyphae by EcO157 can indeed occur, the invaded hyphae may serve as a vessel for enteric bacteria to enter the intracellular space of plants.

### Role of plant susceptibility to downy mildew in EcO157 growth

Reports on the role of plant genotype in the microbial community composition of the lettuce phyllosphere have been inconsistent [26,27]. Numerous lettuce cultivars have been developed in order to minimize crop yield losses incurred from plant disease, including downy mildew. Given the highly hospitable environment of downy mildew lesions to EcO157, as illustrated in Figure 2, we investigated the role of plant susceptibility to *B. lactucae* infection in lettuce colonization by this human pathogen. A commercially released lettuce cultivar Triple Threat and a newly developed lettuce breeding line RH08-0464 were selected to test this hypothesis. Triple Threat is highly susceptible to infection by *B. lactuacea* [28,29], whereas RH08-0464 has high field resistance to this pathogen that is inherited from the stem-type lettuce Balady Banha [28].

EcO157 proliferated to a significantly greater extent in diseased tissue (either in chlorotic or necrotic, or both types of, tissue) than on healthy tissue on both plant accessions (Table 1). Representative discs of healthy, chlorotic, and necrotic lettuce leaf tissue are shown in Figure 1. The extent of EcO157 colonization of the healthy tissue was not dependent on accession (P values ranged between 0.301 and 0.552) in any of the replicate experiments (Table 1). Although we did not attempt to assess specifically the endophytic EcO157 population sizes in our study, this observation is in line with results from a previous study that failed also to detect an overall effect of lettuce cultivar on the degree of *S. enterica* endophytic colonization of leaves, despite a significant role of serovar-cultivar interactions [30]. On the other hand, EcO157 metabolic activity during epiphytic and endophytic colonization of lettuce, as assayed with a *lux* reporter, varied in a cultivar-dependent manner, indicating that plant genotype may affect the human pathogens on the surface or inside healthy leaves [31]. Thus, a range of scenarios remains to be explored regarding the role of cultivar in the contamination of healthy lettuce leaves by enteric pathogens.

**Table 1:** **Effect of romaine lettuce susceptibility to downy mildew on the multiplication of EcO157 in leaf tissue in three replicate experiments**

	**Log-10 increase in EcO157 population size on leaf tissue of two lettuce accessions** ^**a**^								
	**Experiment 1** ^**b**^			**Experiment 2**			**Experiment 3**		
Tissue	RH	TT		RH	TT		RH	TT	
Healthy	1.1 A	1.2 A	(0.301)^c^	0.9 A	0.7 A	(0.420)	0.3 A	0.5 A	(0.552)
Chlorotic	1.1 A	2.5 B	(0.012)	2.1 B	1.8 B	(0.124)	1.2 B	1.3 B	(0.877)
Necrotic	2.8 B	3.2 B	(0.140)	2.4 B	3.1 C	(0.002)	2.3 C	3.1 C	(<0.0001)
	0.0006^d^	0.0015		<0.0001	<0.0001		<0.0001	<0.0001	

^a^Romaine lettuce accessions tested were RH08-0464 (RH) and Triple Threat (TT) with low and high susceptibility to downy mildew, respectively.

^b^Increase in EcO157 population size on the two lettuce accessions was assessed at 18, 20 and 20 h post-inoculation in replicate experiment 1, 2, and 3, respectively.

^c^Values in parenthesis are P values resulting from the t-test between RH and TT for the type of tissue within the experiment.

^d^Values at the bottom of each column are P values resulting from ANOVA on the different types of tissue within plant accession and experiment. Different letters within column indicate means that are significantly different at P < 0.05. Significant differences were determined using the Tukey- Kramer procedure for multiple comparisons.

Importantly, the EcO157 population increase in diseased tissue in our study was significantly lower on lettuce line RH08-0464 than on the more susceptible cultivar Triple Threat in all three replicate experiments (P < 0.05) (Table 1). This suggests that a plant factor linked to the dynamics or amount of tissue infection by *B. lactucae* affects the ability of the human pathogen to fully exploit these lesions for multiplication. The nature of this plant factor remains unclear. It is unlikely that the plant gene(s) that contribute(s) to the resistance of RH08-0464 to *B. lactucae* infection directly inhibited the growth of EcO157 in the lesions because this type of resistance is expected to be specific to *B. lactucae*-lettuce interactions. Recently, chromosomal regions associated with broad-spectrum quantitative disease resistance to plant pathogens were identified [32,33]. It would be of great interest to determine if broad-spectrum disease resistance may be involved not only in plant interaction with plant pathogens but may be of use also to reduce plant colonization by human enteric pathogens.

### Expression of PR-1 in lettuce accessions

Because basal immunity contributes to protection of plants against a broad range of insults, we tested the expression of the gene encoding the antimicrobial PR-1 protein in two lettuce accessions infected with *B. lactucae*. PR-1 is a major marker of the salicylic acid-mediated pathway of basal defense against microbial infection [34] that is induced in plants also in response to oomycete infection [35,36]. Both healthy and chlorotic leaf tissue were tested for PR-1 expression in the resistant line RH08-0464 and in the susceptible cultivar Triple Threat. Necrotic lesions do not yield sufficient mRNA to allow for transcript measurement by qRT-PCR.

**Figure 4:**
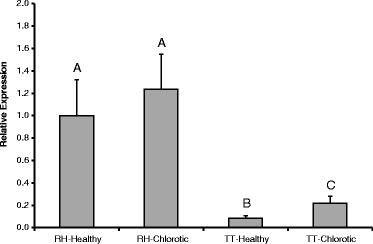
**Quantification by qRT-PCR of the transcriptional activity of the plant defense response gene PR-1 in healthy and chlorotic tissue of leaves infected with** ***B. lactucae*** **in the resistant line RH08-0464 (RH) and the susceptible cultivar Triple Threat (TT).** The data illustrate mean expression of PR-1 in three replicates relatively to its activity in the healthy tissue of RH08-0464, which was set to a value of 1. RH-Healthy and –Chlorotic, indicates PR-1 expression in healthy and chlorotic tissue of line RH08-0464, respectively; TT-Healthy and –Chlorotic, indicates PR-1 expression in healthy and chlorotic tissue of cultivar Triple Threat, respectively. PR-1 expression data was normalized to that of the ACT7 gene across samples. Error bars indicate standard error of the mean. Different letters indicate means that are significantly different at P < 0.05.

qRT-PCR analysis revealed that accession RH08-0464 had significantly greater expression of PR-1 than cultivar Triple Threat (Figure 4). The high expression of this gene in the healthy RH08-0464 tissue is likely caused by its systemic induction, since PR-1 is the hallmark of systemic acquired resistance [34]. Thus, because the healthy tissue was sampled from leaves that harbored lesions at various stages of the disease, the defense signal induced by the oomycete actively infecting the leaf at other distant sites likely resulted in production of PR-1 in the healthy part of the same leaf. This accumulation of PR-1 may not have affected the growth of the human pathogen on the healthy tissue of the tested accessions since most, if not all, of the bacterial cell population was located on the leaf cuticle and therefore, did not have open access to the plant apoplast as did occur in the lesions. The general trend of PR-1 transcriptional activity agrees with a potential role of *B. lactucae*-induced basal immunity in the weaker EcO157 colonization of lesions in RH08-0464. A different but not necessarily contradictory hypothesis, may be that EcO157 benefited from an immuno-suppressive effect of *B. lactucae* on the cultivar Triple Threat, thereby experiencing an environment that enhanced its growth compared with the lesions in the more resistant RH08-0464. This type of symbiotic interaction was observed with co-inoculations of the plant pathogenic *P. syringae* pv. *tomato* DC3000Δ*hopQ1-1* and *S. enterica* in *Nicotiana benthamiana* [37].

### Lesion-mediated protection of EcO157 from desiccation

**Figure 5:**
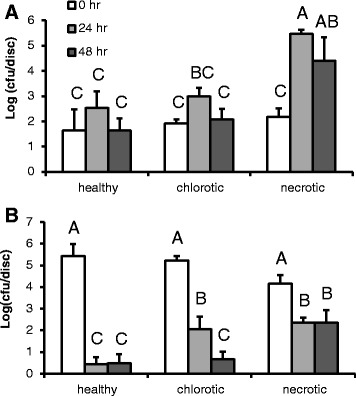
**EcO157 population dynamics on romaine lettuce leaves (cultivar Green Towers) over time after inoculation and incubation of the leaves under conditions of warm temperature and 90-100% RH (A) or 65-75% RH (B).** Population sizes of EcO157 were assessed on leaf discs of healthy, chlorotic, and necrotic tissue of *B. lactucae*-infected leaves immediately (white bars), at 24 h (gray bars), and 48 h (black bars) post-inoculation. Data points represent the mean of log-10 (cfu per disc) for six (high RH) and eight (low RH) replicate discs sampled from different leaves. Error bars indicate standard error of the mean. Different letters within panel indicate means that are significantly different at P < 0.05. Note that leaves at 65-75% RH were inoculated with greater concentrations of EcO157 than leaves maintained at 90-100% RH to allow for quantification of survival rate.

Bacterial cells in the phyllosphere frequently encounter periods of dry conditions when free water is not available on the plant surface [38]. Given that EcO157 cells can become embedded in the downy mildew-infected tissue and that the physicochemical properties of the damaged tissue itself may alter the microenvironment and the physiology of the human pathogen, we sought to determine if EcO157 cells gained protection from desiccation stress on lettuce leaves by localization in *B. lactucae* lesions. Our experimental set up, using cultivar Green Towers, simulated high relative humidity conditions (90-100% RH) where free water remained at few locations on the leaves at the macroscopic level, and conditions of 65-75% RH where free water was absent from the leaf surfaces macroscopically. EcO157 multiplied over the first 24 h after inoculation on healthy, chlorotic, and necrotic tissue under high RH, with the greatest increase observed in the necrotic lesions (Figure 5A). The population sizes then decreased between 24 and 48 h post-inoculation, possibly because a certain proportion of the wet sites on the leaves dried up over time after inoculation. At the lower RH of 65-75%, EcO157 populations declined on all types of tissue over time but remained proportionally the greatest in the necrotic lesions with a survival rate at 48 h post-inoculation of 1.58% compared with 0.0011% and 0.0028% in the healthy and chlorotic parts of the leaf, respectively (Figure 5B). It is noteworthy that under the lower RH, the EcO157 cell numbers appeared to stabilize over time after an initial decrease in the necrotic tissue, suggesting that the human pathogen adapted to the conditions in that habitat either physiologically or by reaching protective niches. Contribution of the bacterial plant pathogen, *Xanthomonas campestris* pv. *vitians* to the persistence of EcO157 on lettuce in the field, where low RH and water availability on the phylloplane may be important stressors during various times of the day has been reported [3].

Our study is the first to determine the effect of downy mildew, an endemic disease of lettuce in a major vegetable production region of the USA, on the behavior of EcO157 on this crop and to assess various factors that may enhance its persistence in infected leaves. Given the great potential for multiplication and survival of EcO157 in *B. lactucae* lesions on wet and dry leaves, respectively, and considering that large fluctuations in water availability to bacterial cells likely prevail on lettuce in the field, downy mildew disease may represent an important risk factor of microbial contamination. The higher prevalence of downy mildew on lettuce plants approaching harvest maturity may compound the risk of contamination associated with this disease. Although most of the old lettuce leaves infected with downy mildew are trimmed off during harvest, a fraction of *B. lactucae* lesions escape visual detection. Additionally, the proliferation and enhanced survival of EcO157 in the infected tissue may create a reservoir from which the pathogen is disseminated or vectored to other sites in the field, as well as contribute to the persistence of the human pathogen in crop debris in the field. Further investigation of the role of downy mildew in the colonization of lettuce by enteric pathogens under field conditions is warranted. Additionally, our observation that EcO157 survives and multiplies at different rates in comparable *B. lactucae* lesions of two accessions indicates that variability in lettuce gene pool may be explored to develop cultivars that are less hospitable to human enteric pathogens. Such cultivars would be a vital part of an integrated strategy to minimize EcO157 illness linked to lettuce contamination.

## Conclusions

The massive proliferation of EcO157 (and *S.* Typhimurium) under wet conditions in downy mildew lesions on lettuce leaves, and its enhanced persistence in this diseased tissue under conditions of lower RH, suggest that infection of lettuce by *B. lactucae* may increase the risk of microbial contamination of this crop. Given that differences in EcO157 colonization of diseased tissue were observed in two lettuce accessions with different levels of resistance to downy mildew, breeding lettuce for resistance to this disease may be worth exploring to decrease the burden of enteric illness due to contamination of lettuce.

## Methods

### Strains and media

*E. coli* serovar O157:H7 strain ATCC 43888, a natural strain that does not produce shiga toxin 1 and 2, and *S. enterica* serotype Typhimurium strain SL1344, were used in this study. A spontaneous rifampin-resistant strain of *E. coli* O157:H7 43888 was obtained by selection on Luria Bertani (LB) amended with rifampin at 100 μg/ml. The spontaneous mutant and parental strain were compared in LB throughout all phases of culture and did not show any difference in fitness. *S.* Typhimurium strain SL1344 is naturally resistant to streptomycin. The strains were grown to stationary phase at 28°C in Luria Bertani broth - half salt (0.5% NaCl), the cells were washed twice in potassium phosphate buffer (KP) (10 mM, pH 7.0), and then resuspended in KP buffer (1 mM, pH 7.0) at the cell concentration specified below for each experiment.

### Plant materials

Lettuce (*Lactuca sativa* L.) plants were grown in the field at the USDA, ARS experimental station in Salinas, CA. The crop was cultivated using standard cultural practices for the area except that no fungicide treatment was applied in order to allow for natural infection by *B. lactucae* and development of downy mildew. At harvest maturity, when a full head had formed, naturally infected plants were transplanted into plastic pots (27 cm in diameter, 24 cm height) and transported to the USDA, ARS in Albany, California for experimentation. Two cultivars and one breeding line of romaine lettuce with different levels of field resistance to *B. lactucae* were used in experiments: susceptible cultivar Triple Threat with a low level of resistance, cultivar Green Towers with an intermediate level of resistance, and breeding line RH08-0464 with a high level of resistance [28,29]. Green Towers is one of the main cultivars used for commercial production in the USA. Because naturally occurring infection was used, races of *B. lactucae* that infected the plants were not controlled for. However, based on recent data from the same lettuce growing area, the most frequently detected avirulence genes in *B. lactucae* were *Avr17*, *Avr37*, *Avr38*, and *Avr36* [12].

### Inoculation and incubation of inoculated lettuce leaves

Immediately before inoculation with enteric pathogens, leaves that had symptoms of downy mildew at the chlorotic and necrotic stages of the disease were harvested from the potted plants. Each replicate detached leaf was inoculated individually by holding it at its base and immersing it upside down in the inoculum suspension for 3 sec and then briefly draining the excess inoculum suspension, as we previously described [17].

Comparison of EcO157 and *S.* Typhimurium proliferation on healthy and diseased lettuce tissue was conducted only on the cultivar Green Towers and under conditions of free water on the leaf surface in order to assess the overall effect of downy mildew on their colonization of lettuce; all other experiments in this study were performed with EcO157 only. For comparison of *S.* Typhimurium and EcO157, the leaves were inoculated with single strains in a suspension of 10^6^ cells/ml. For comparison of colonization of various lettuce accessions by EcO157, leaves were also inoculated in a suspension of 10^6^ cells/ml. In both types of experiments, the leaves were then incubated horizontally in a single layer in a large plastic tub lined with wet paper towels and covered with a plastic bag, which promoted the presence of free water on the leaves. The tub was then placed in an incubator at 28°C. This experimental set up was devised to test the maximum growth potential of the enteric pathogens on healthy tissue, and on chlorotic and necrotic tissue due to infection by *B. lactucae*.

In order to investigate the effect of relative humidity on the persistence of EcO157 on healthy and diseased tissue, the leaves were inoculated in a suspension of 2 × 10^5^ and 10^7^ cells/ml for incubation under high and low relative humidity (RH), respectively. Then, the lettuce leaves were incubated upright in a small container with water at the bottom to maintain leaf turgidity. Half of the containers were covered with a plastic bag tightened with a rubber band around the bottom of the container and the other half remained uncovered. All of the containers were placed in a chamber maintained at 28°C and 65-75% RH. This set up resulted in the leaves in the covered containers being exposed to 90-100% RH, whereas those in the uncovered containers experienced 65-75% RH. By the end of the experiment, free water was absent macroscopically on the leaves under low RH, whereas leaves under high RH still harbored free water at rare locations. RH and temperature in the chamber were monitored with a Dickson TH8P5 Chart Recorder equipped with a remote sensor (Dickson, Addison, IL). Four, eight and six replicate discs were used in the first, second and third replicate experiment, respectively.

### Recovery of bacterial cells from leaf tissue and population measurement

Immediately after inoculation and at indicated times, discs 9 mm in diameter of healthy, chlorotic, and necrotic tissue, as illustrated in Figure 1 were sampled from the inoculated leaves with a cork borer #5. Sampling was performed at random from a total of 15 replicate leaves; the three types of tissue were not necessarily taken from the same leaf. The number of replicate discs varied from three to eight depending on the type of experiment and is indicated in figure legends.

To recover bacteria from leaf tissue, each disc was homogenized with a mortar and pestle in 2 ml KP buffer (10 mM, pH 7.0). The homogenate and dilutions were plated onto LB agar containing rifampin (100 μg/ml) or streptomycin (30 μg/ml) for bacterial counts of EcO157 and *S. enterica*, respectively. For experiments under low RH conditions and in which the population size of EcO157 declined over time, the entire disc homogenate was plated and thus, the detection limit for measurement of population size was one cell per disc of leaf tissue. The plates were incubated at 37°C overnight and colonies counted to assess population sizes per disc of leaf tissue.

### Microscopy

Small discs of healthy and necrotic lettuce tissue were sampled from the leaves at 48 h post-inoculation as described above and mounted with AquaPolymount (Polysciences, Warrington, PA) for visualization with a Leica SP5 confocal microscope (Leica Microsystems, Wetzlar, Germany). The green fluorescent signal was obtained from GFP-EcO157 43888 transformed with pGT-KAN, a stably maintained plasmid expressing *gfp* constitutively from the kanamycin resistance gene promoter [39], which was used in EcO157 in our previous studies [17,40]. The red fluorescent signal was obtained from the autofluorescence of *B. lactucae* and from the chloroplasts of the leaf tissue. In one instance, the far-red autofluorescent signal of the *B. lactucae* hyphae was pseudocolored in blue in order to better distinguish them from the GFP-EcO157 cells that colonized some of the hyphae infecting the leaves.

### qRT-PCR

Expression of the basal plant defense gene PR-1 was quantified in romaine lettuces Triple Threat and RH08-0464. Discs from uninoculated and nonincubated leaves with natural infection of *B. lactucae* from the field were harvested from the same plants that were sampled for leaf inoculation. The discs were frozen in liquid nitrogen and stored in the −80°C freezer until used for qRT-PCR. Six discs from each type of tissue were sampled at random from ten leaves. The discs were ground individually using a mortar and pestle in liquid nitrogen before RNA was extracted using the Ambion® TRIzol® reagent and PureLink® RNA kit (Life Technologies, Carlsbad, CA). The RNA was tested for absence of DNA and analyzed by qRT-PCR with the Stratagene Brilliant II SYBR Green kit as described previously [22,41]. Expression of ACT7 was used to normalize the data between samples based on the method by Pfaffl [42]. The sequences of the primers, based on *Lactuca sativa* nucleotide sequences in GenBank, were: PR-1-F 5′TCGCCACAAGACTTTGTTAATG, PR-1-R 5′GAGGCAAGATTTTCACCATAGG, ACT7-F 5′GCAATTCAAGCCGTTCTTTC, and ACT7-R 5′GATCCAAACGGAGGATAGCA.

### Statistical analysis

All experiments were replicated at least twice with plants harvested from the field at different times. Analysis of variance (ANOVA), *t*-test, and descriptive statistical analyses were performed with the software JMP v. 11.1.1 (SAS Institute, Cary, NC, USA). Significant differences for multiple comparisons were determined using the Tukey-Kramer HSD (honest significant difference) procedure.
